# Inoculum selection and hydraulic retention time impacts in a microbial fuel cell treating saline wastewater

**DOI:** 10.1007/s00253-024-13377-y

**Published:** 2025-01-28

**Authors:** Antonio Castellano-Hinojosa, Manuel J. Gallardo-Altamirano, Clementina Pozo, Alejandro González-Martínez, Jesús González-López

**Affiliations:** 1 Environmental Microbiology Group, Institute of Water Research, University of Granada, 18003 Granada, Spain; 2 Department of Microbiology, University of Granada, 18071 Granada, Spain; 3 Department of Chemical Engineering, University of Granada, 18071 Granada, Spain

**Keywords:** Voltage production, MFC, Salinity, Wastewater, Electroactive microorganisms

## Abstract

**Abstract:**

Microbial fuel cell (MFC) technology has received increased interest as a suitable approach for treating wastewater while producing electricity. However, there remains a lack of studies investigating the impact of inoculum type and hydraulic retention time (HRT) on the efficiency of MFCs in treating industrial saline wastewater. The effect of three different inocula (activated sludge from a fish-canning industry and two domestic wastewater treatment plants, WWTPs) on electrochemical and physicochemical parameters and the anodic microbiome of a two-chambered continuous-flow MFC was studied. For each inoculum, three different HRTs were tested (1 day, 3 days, and 6 days). The inoculum from the fish canning industry significantly increased voltage production (with a maximum value of 802 mV), power density (with a maximum value of 78 mW m^−2^), coulombic efficiency (with a maximum value of 19.3%), and organic removal rate (ORR) compared to the inocula from domestic WWTPs. This effect was linked to greater absolute and relative abundances of electroactive microorganisms (e.g., *Geobacter*, *Desulfovibrio*, and *Rhodobacter*) and predicted electron transfer genes in the anode microbiome likely due to better adaption to salinity conditions. The ORR and current production were also enhanced at shorter HRTs (1 day vs. 3 and 6 days) across all inocula. This effect was related to a greater abundance and diversity of bacterial communities at HRT of 1 day compared to longer HRTs. Our findings have important bioengineering implications and can help improve the performance of MFCs treating saline effluents such as those from the seafood industry.

**Key points:**

*• Inoculum type and HRT impact organic matter removal and current production.*

*• Changes in bioenergy generation were linked to the electroactive anodic microbiome.*

*• Shorter HRT favored increases in the performance of the MFC.*

**Graphical Abstract:**

**Figure:**
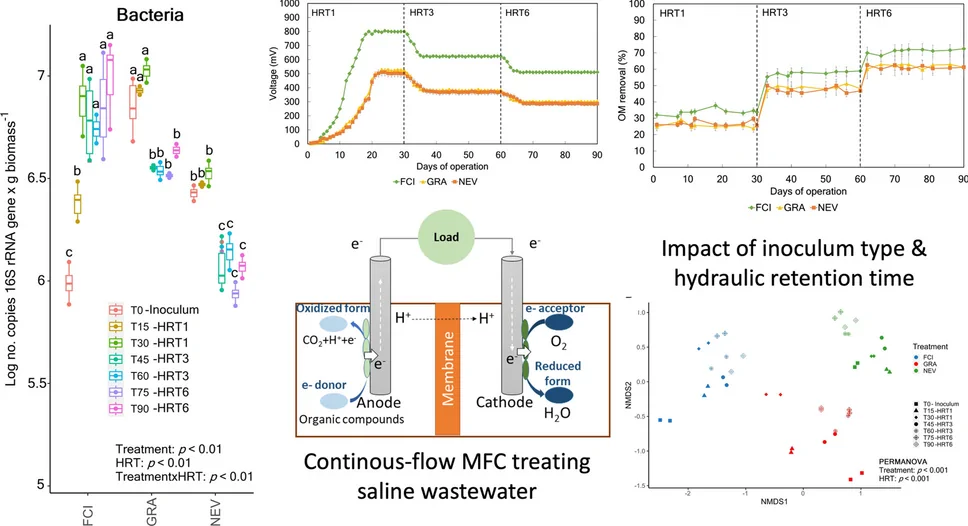

**Supplementary Information:**

The online version contains supplementary material available at 10.1007/s00253-024-13377-y.

## Introduction

Microbial fuel cells (MFCs) have received increased interest as a promising and sustainable approach for treating domestic and industrial wastewater (Boas et al. 2022; Tsekouras et al. 2022). This technology not only facilitates the removal of organic compounds but also produces electricity from liquid waste, making it a significant advancement in wastewater treatment (Kumar et al. 2022; Saravanan et al. 2023). However, uncertainties regarding economic viability and energy production have hindered the scale-up and commercialization of MFCs (Jadhav et al. 2021; Tsekouras et al. 2022). While previous research has shown the efficacy of novel bioelectrochemical systems in desalinating saline wastewater (Odunlami et al. 2023), less attention has been directed toward investigating the potential of MFCs for simultaneously removing organic contaminants and generating electricity from saline wastewater (Kim and Logan 2013; Kumar et al. 2022; Saravanan et al. 2023; Castellano-Hinojosa et al. 2024).

The seafood processing industry generates significant volumes of wastewater during various production stages, including cleaning, chilling, blanching, filleting, cooking, and marination. Estimates suggest that the treatment of 1 ton of seafood requires 10 to 40 cubic meters of water (Arvanitoyannis and Kassaveti 2008; Venugopal 2021). This wastewater is characterized by moderate levels of organic compounds (ranging from 0.4 to 2 g L^−1^ of total organic carbon, TOC) and salinity (ranging from 1.2% to 10%), posing unique challenges for treatment and distinguishing it from other industrial effluents (Lefebvre and Moletta 2006; Chowdhury et al. 2010; Venugopal and Sasidharan 2021; Correa-Galeote et al. 2021a). Previous research has demonstrated the potential of saline industrial and domestic wastewater to support bioenergy generation in MFCs due to their high conductivity (Lefebvre et al. 2012; Li et al. 2013; Guo et al. 2021). A significant limitation in the biological remediation of saline wastewater persists because microorganisms have a restricted capacity to adjust their metabolism to elevated salinity levels (Lefebvre and Moletta 2006). Recent investigations have indicated that inoculation of continuous-flow MFCs with halophilic bacteria can achieve chemical oxygen demand (COD) removal rates exceeding 90% with a maximal power density of 420 mW m^−2^ using industrial saline wastewater (Jamal and Pugazhendi 2021). However, other studies have reported similar performance in MFCs operated in batch mode and inoculated with activated sludge from domestic wastewater treatment plants (WWTPs) where microbial communities were not acclimated to elevated saline conditions (Li et al. 2013; Guo et al. 2021; Xin et al. 2022; Sibi et al. 2023). These contrasting results could be due to differences in salinity levels and MFC design and operation between studies, which may impact anode colonization and subsequent energy production and organic matter (OM) removal efficiency. Continuous-flow MFCs are better suited for practical applications than batch-mode MFCs because they can handle larger volumes and operate for extended durations (Cai et al. 2014; Cabrera et al. 2022). Moreover, much of the research on saline wastewater in MFCs has been conducted using fed-batch mode for short periods, typically between 6 to 24 h (Cai et al. 2014; Cabrera et al. 2022). Yet, additional information is needed on the effect of the inoculum source on electrochemical, physicochemical, and microbial parameters in MFCs fed with saline wastewater and operated in continuous mode.

The hydraulic retention time (HRT) plays a critical role in the performance of MFCs (Ye et al. 2020; Tanaka et al. 2024; Castellano-Hinojosa et al. 2024). In general, previous research indicates that higher HRTs tend to enhance OM removal and increase electricity production in MFCs (Kim et al. 2015, 2016; Sobieszuk et al. 2017; Fazli et al. 2018; Ye et al. 2020; Castellano-Hinojosa et al. 2024). However, increases in current generation with lower HRT have been reported (Juang et al. 2012; Wei et al. 2012; Akman et al. 2013; Ge et al. 2013). Additionally, HRT impacts the level of shear stress experienced by the electroactive biofilm formed on the anode surface, which directly influences its formation (Lecuyer et al. 2011; Sorgato et al. 2023). Further research is imperative to comprehensively understand the impact of varying HRTs and inocula on MFC performance when treating saline wastewater, potentially aiding in selecting optimal operational conditions to maximize energy production and OM removal.

Exoelectrogenic microorganisms play a pivotal role in MFCs (Logan et al. 2019; Lovley and Holmes 2021). They directly generate electrical currents by oxidizing OM within the anode chamber under oxygen-limiting conditions, transferring electrons to an electrode via various mechanisms (Koch and Harnisch 2016; Logan et al. 2019; Castellano-Hinojosa et al. 2022a, b). In continuous-flow MFC systems operating at short HRTs, direct conductive species become particularly crucial. This is because soluble mediators and planktonic cells are swiftly carried away, contrasting with batch culture MFCs (Pannell et al. 2016; Logan et al. 2019). Previous research has shown that the study of the anodic microbiome can help to understand treatment efficiency and current generation in MFCs operated in continuous mode (Lovley and Holmes 2021; Castellano-Hinojosa et al. 2022a, b; Jalili et al. 2024). However, studies on the impact of activated sludge selection and HRT on bioenergy generation and the abundance, diversity, and community composition of electroactive microorganisms in MFCs treating saline wastewater are scarce.

In this work, the impact of three different inocula on OM removal %, current production, and the anodic microbiome (abundance, diversity, and community composition) was explored in a continuous-flow MFC fed with saline wastewater. For each inoculum, the MFC was operated at three HRTs to further understand the combined effect of the inoculum source and HRT on the physicochemical, electrochemical, and microbial parameters. The impact of the inoculum type and the HRT on the relative abundance of predicted genes involved in electron transfer were also examined. This study contributes to elucidating the impact of the inoculum type and HRT on physicochemical and electrochemical parameters, and microbial communities of MFCs treating saline wastewater. It was hypothesized that inocula previously adapted to saline conditions would result in higher current production and OM removal efficiency due to a greater abundance and diversity of electroactive microorganisms. It was also hypothesized that higher HRTs would enhance the efficiency of OM removal and energy production regardless of the inoculum source with effects on the anode microbiome.

## Materials and methods

### Description and operation of the MFC system

A two-chambered continuous-flow MFC reactor (H-cell type) was used in this study (Supplementary Fig. S1). The MFC consisted of two chambers of methacrylate with an anode (5 L) and a cathode (4 L). These compartments were divided by a proton exchange membrane (Nafion N117, Chemours, Italy), pre-treated following the manufacturer's instructions. The anode was constructed using carbon fibers (6.35 mm thickness; 240 cm^2^ projected area), while the cathode was fashioned from a copper bar (17 cm^2^ projected area). Both chambers were connected using a copper conductor cable for electron transport. Sensors for measurements of redox potential, pH, temperature, and dissolved oxygen (DO) concentrations were placed in both chambers (Supplementary Fig. S1).

Three different inocula were tested in this study. The anode was separately inoculated with 1) activated sludge from a fish-canning industry in Galicia (Galicia, Spain, located at 100 m.a.s.l.) (FCI treatment); 2) activated sludge from a domestic WWTP (Los Vados, Granada, located at 670 m.a.s.l.) (GRA treatment); and 3) activated sludge from a domestic WWTP located near Sierra Nevada ski resort in a high-mountain area (Granada, Spain, located at 2000 m.a.s.l.) (NEV treatment). The three WWTPs used a conventional activated sludge system. The WWTP for the treatment of canning food operates at a moderate salinity level (around 12–20 g NaCl L^−1^; Correa-Galeote et al. 2021a) while the domestic WWTPs operate at low salinity (< 2 g NaCl L^−1^). Before inoculation, the biomass concentration in the different inocula was measured via mixed liquor-suspended solids (MLSS). The anode chamber was inoculated with 4 L of the different inocula, which was diluted to a final concentration of 2.6 g/L. This biomass concentration was estimated based on previous experiments (Castellano-Hinojosa et al. 2024). Before starting the experiment, the inocula were recirculated for 4 days at 4 L h^−1^ and continuously mixed using a magnetic stirrer at 1500 rpm to favor anode colonization. This time period was estimated enough based on visual observation of the biomass on the anode and residual concentration of suspended solids in the water (less than 2 mg L^−1^). For each inoculum, three consecutive HRTs were run for 30 days: 1 day (HRT1), 3 days (HRT3), and 6 days (HRT6). The MFC was properly cleaned between inoculations, and the carbon fibers of the anode chambers were replaced with new ones.

The anode chamber was fed with a synthetic medium simulating wastewater from the fish-canning industry (Correa-Galeote et al. 2021b): CH_3_COONa 3H_2_O 5.6 g L^−1^ (2.5 g Ac^−^/L), K_2_HPO_4_ 0.085 g L^−1^, NaCl 5.23 g L^−^1, KH_2_PO_4_ 0.03 g L^−1^, NH_4_Cl 0.38 g L^−1^, KCl 0.04 g L^−1^, MgSO_4·_7H_2_O 0.1 g L^−1^, pH 7.1, and conductivity 13.8 mS cm^−1^. The influent's levels of total organic carbon (TOC) and sodium chloride (NaCl) were adjusted to 1 and 10 g L^−1^, respectively, to replicate typical concentrations found in wastewater from fish canning facilities (Correa-Galeote et al. 2021a, b). The organic loading rate (OLR) varied across different HRTs, specifically 2.5 g COD L^−1^ d^−1^ at HRT1, 0.8 g COD L^−1^ d^−1^ at HRT3, and 0.4 g COD L^−1^ d^−1^ at HRT6. The catholyte received a phosphate buffer (Rossi and Logan 2020), which was replaced every 2 weeks. Air was continuously bubbled through the catholyte at a rate of 8.6 mg L^−1^. Both the anode and cathode chambers were kept mixed using a magnetic stirrer rotating at 1500 rpm. The MFC system was maintained at 22 °C within a controlled-temperature chamber.

### Electrochemical analyses

Current production (mW), current density (mA m^−2^ of anode), power density (mW m^−2^ of anode), and coulombic efficiency (CE) were measured as described earlier (Castellano-Hinojosa et al. 2024). The continuous-flow MFC system was operated with an external resistance of 1000 Ω which was selected based on preliminary studies.

### Physiochemical analyses

Influent and effluent samples (500 mL) for physicochemical analyses were collected twice per week. The organic removal rate (ORR) per day was estimated by subtracting the COD in the influent from that in the effluent and then dividing by the corresponding HRT. The COD, MLSS, and suspended solids concentrations were determined using standard protocols (APHA 2012). Acetate (CH_3_‒COO^−^), ammonium (NH_4_^+^), nitrite (NO_2_^−^), and nitrate (NO_3_^−^) were quantified using an ion chromatograph (Metrohm Ion Chromatograph, AG, Switzerland). The organic removal % was estimated by subtracting the concentration of CH_3_‒COO^−^ in the influent from that in the effluent. Temperature, pH, and redox potential in the anode were continuously monitored using sensors positioned in the chamber.

### Extraction of DNA and quantification of prokaryotic communities

Anode biomass was collected in duplicate after 0 (original inoculum), 15, and 30 days of operation for each inoculum and HRT. The biofilm on the anode was removed using sterilized tweezers and placed in 30 mL of sterilized saline solution (0.9% NaCl). After that, the samples underwent sonication for 3 min and centrifugation at 13000 rpm for 5 min. The resulting biomass was stored at −20ºC until needed. DNA extraction and quantification were carried out as described in Castellano-Hinojosa et al. (2024).

To determine the abundance of bacterial (16SB) and archaeal (16SA) communities in the anodic microbiome, a QuantStudio 3 Real-Time thermocycler (ThermoFisher, USA) was utilized. The reaction mixtures, conditions, primers, and standards used for PCR amplifications were detailed by Castellano-Hinojosa et al. (2024) and are provided in Supplementary Table S1. The standard curves exhibited linearity (R^2^ > 0.994), and the amplification ranged from 91.2% to 99.3%.

### Sequencing analysis of prokaryotic communities and bioinformatics

The prokaryotic community (Bacteria and Archaea) was amplified via PCR using the primer pairs Pro341F and Pro805R (Takahashi et al. 2014). Subsequently, the samples were sequenced utilizing an Illumina MiSeq sequencer at Novogene Europe (Cambridge, UK). Raw reads underwent processing in QIIME2 following the methodology described by Castellano-Hinojosa et al. (2021). The resulting dataset contained an average of 55,124 sequences per sample. Raw sequence data can be accessed through the NCBI under BioProject PRJNA1102839.

The study of alpha diversity (including the number of ASVs, Shannon index, and Inverse Simpson index) and beta diversity was conducted using the "vegan" and “Phyloseq” packages in R. Changes in the community composition across treatments and time points were assessed through PERMANOVA analysis in R. Genera exhibiting statistical differences in their relative abundance between time points were identified employing the “DESeq2” package in R (Castellano-Hinojosa et al. 2022a, b). Significant Pearson correlations between the relative abundance of differentially abundant taxa, OM removal %, and current production were determined using the “cor.test()” function in R. The PICRUSt2 tool (Douglas et al. 2020) was used to predict the relative abundance of KEGG orthologs (KOs) involved in the electron transfer pathway as described by Castellano et al. (2022a, b).

### Statistical analysis

All statistical analyses were done in R software version 4.1.2. (http://www.rproject.org/). Normality and homoscedasticity assumptions using the Shapiro–Wilk test and Bartlett's tests, respectively. Significant differences among treatments and HRTs for the physicochemical and microbial variables were tested by linear mixed-effects (LME) model with treatment, HRT, and their interactions considered as fixed factors using the ‘lme4’ library of R (Bates et al. 2015). The effect of the physicochemical and electrochemical properties as abiotic factors controlling the changes in the absolute abundances of bacterial and archaeal communities were also assayed by LME model. Data from all treatments were considered together for the models. The “treatment”, “HRT”, and their interaction were considered as random factors. Before model execution, multicollinearity among continuous predictors was checked. Significant effects were determined through ANOVA (*p*-value), and the normality of the models' residuals was checked using the Shapiro–Wilk test. Differences among the levels of random categorical factors were assessed using Tukey's post hoc tests, implemented with the ‘lsmeans’ package in R.

## Results

### Effect of inoculum type and HRT on electrochemical parameters

**Fig. 1:**
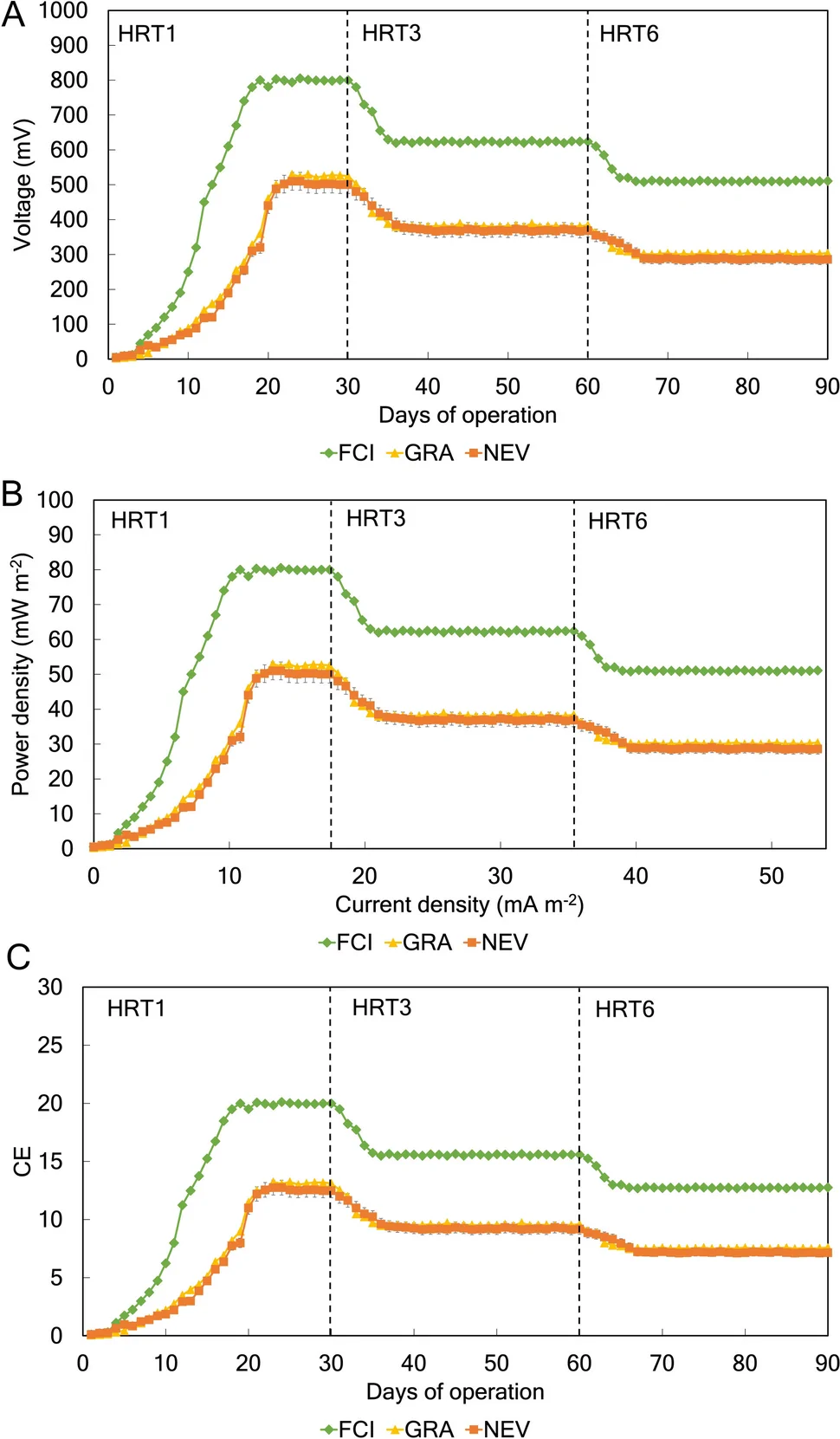
Voltage production (**A**) power density as a function of current density (**B**), and CE (**C**) during the experimental period. The MFC was inoculated with activated sludge from a fish-canning industry (FCI) and two different domestic WWPTs (GRA and NEV). Three consecutive HRTs were examined for each treatment: 1 day (HRT1), 3 days (HRT3), and 6 days (HRT6). CE, coulombic efficiency

The inoculum type and the HRT determined variations in energy production in an MFC fed with saline wastewater and operated in continuous mode. Regardless of the HRT, the use of the inoculum from the fish-canning industry (FCI) produced the greatest values for voltage production (with a maximum value of 802 mV; Fig. 1A), power density (with a maximum value of 78 mW m^−2^; Fig. 1B), and CE (with a maximum value of 19.3%; Fig. 1C) compared to the inoculum from domestic WWTPs. No differences in voltage production (with maximum values of 495 mW and 488 mW, respectively), power density (with maximum values of 49.2 mW m^−2^ and 48.6 mW m^−2^, respectively), and CE (with maximum values of 13.5% and 13.2%, respectively) were observed between the GRA and NEV treatments (Fig. 1). Regardless of the inoculum, the greatest values of voltage production (Fig. 1A), power and current densities (Fig. 1B), and CE (Fig. 1C) were detected when the MFC was operated at HRT1 compared to HRT3 and HRT6. Decreases in energy production were observed with increased HRT from 1 to 3 days, and from 3 to 6 days, respectively (Fig. 1).

### Effect of inoculum type and HRT on physicochemical parameters

**Fig. 2:**
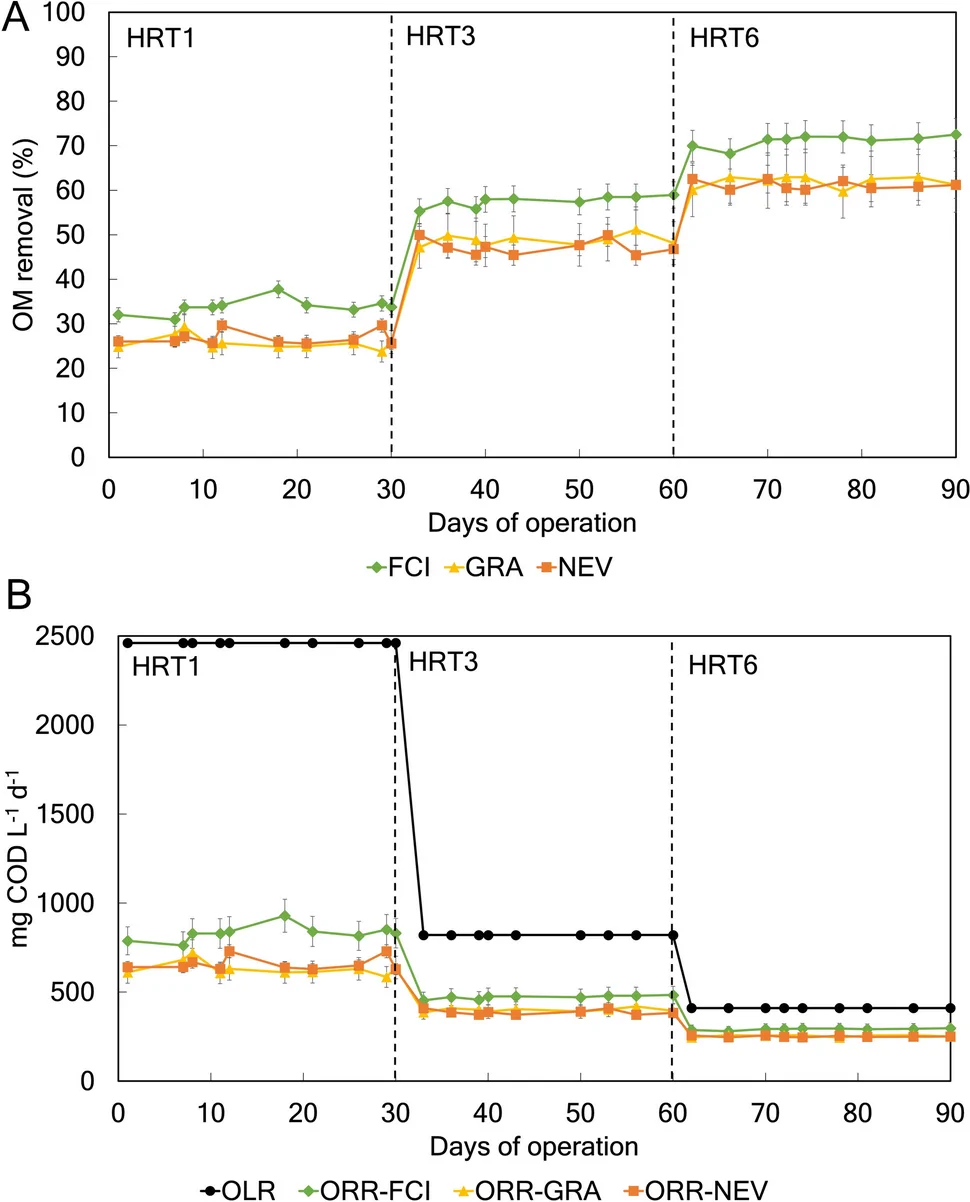
OM removal % (**A**) and ORR and ORL (**B**) during the experimental period. The MFC was inoculated with activated sludge from a fish-canning industry (FCI) and two different domestic WWPTs (GRA and NEV). Three consecutive HRTs were examined for each treatment: 1 day (HRT1), 3 days (HRT3), and 6 days (HRT6). OM, organic matter; ORR, organic removal rate. ORL, organic loading rate. Values are expressed as mean with standard error

The inoculum type and the HRT had a significant effect on the OM removal % and ORR (Fig. 2). Regardless of the HRT, values of OM removal % were significantly greater in the MFC inoculated with FCI compared to GRA and NEV (Fig. 2A). No differences in OM removal % were observed between the GRA and NEV treatments irrespective of the HRT (Fig. 2A). Regardless of the inoculum, increases in OM removal % were observed at higher HRT. The ORR significantly decreased with longer HRT and values were always significantly greater in the MFC inoculated with FCI compared to GRA and NEV (Fig. 2B). Regardless of the HRT, no significant differences in the ORR were observed between the GRA and NEV treatments (Fig. 2B).

Treatment and HRT had no significant impact on the pH, temperature, redox potential of the anode, concentration of suspended solids in the effluent, and N removal % (Supplementary Table S2). The FCI, GRA, and NEV treatments had slightly different original pH values (7.6 ± 0.2, 7.3 ± 0.2, and 7.2 ± 0.1, respectively). Temperature and redox potential of the anode, and N removal % remained stable 5 days post-inoculation and were in the range of 21.1–21.6 ºC, −467 to −494 mV, and 26.3–39.4, respectively. The concentration of suspended solids in the effluent tended to be significantly greater during the first 5–8 days post-inoculation (in the range of 24–31 mg L^−1^, 12–24 mg L^−1^, and 8–18 mg L^−1^ for the FCI, GRA, and NEV treatments) to decrease afterward and remain stable during the rest of the experiment.

### Abundance of prokaryotic communities and their controlling drivers

**Fig. 3:**
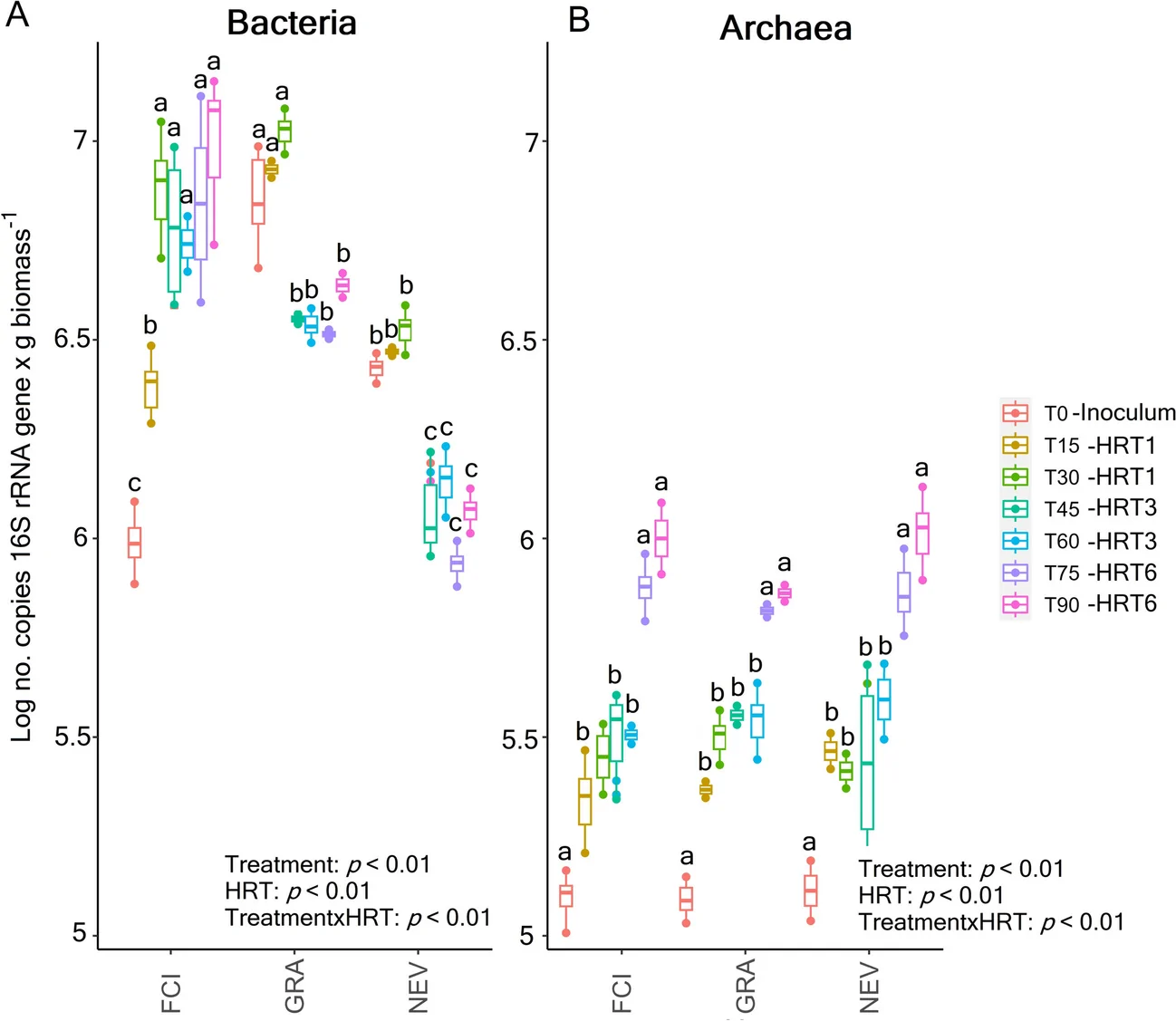
Absolute abundance of bacterial (16SB, **A**), archaeal (16SA; **B**) communities at different time points during the experimental period. The MFC was inoculated with activated sludge from a fish-canning industry (FCI) and two different domestic WWPTs (GRA and NEV). Three consecutive HRTs were examined for each treatment: 1 day (HRT1), 3 days (HRT3), and 6 days (HRT6). Different letters above the bars indicate significant differences between time points. Linear mixed-effects model together with Tukey's post hoc test were used to look for significant differences between treatments and HRTs (*p* < 0.05). Values are expressed as mean with standard error

Treatment, HRT, and their interaction had a significant effect on the absolute abundance of bacterial (Fig. 3A) and archaeal (Fig. 3B) communities in the anode biofilm. Treatment with FCI significantly increased the abundance of 16SB communities until day 30 of operation at HRT1 to remain unchanged at HRT3 and HRT6 until the end of the experiment. When the MFC was treated with GRA and NEV, the abundance of 16SB communities remained unchanged post-inoculation until day 30 of operation at HRT1. Then, it was significantly reduced at HRT3 and HRT6 towards the end of the experiment with no differences between these two HRTs. Regardless of the treatment, gradual and significant increases in the abundance of 16SA communities were observed post-inoculation at HRT1 with no differences compared to HRT3. The total abundance of the 16SA community was significantly greater at HRT6 compared to HRT3 for all treatments.

Linear mixed effects models revealed the effects of the physicochemical and electrochemical properties as abiotic factors that control changes in the absolute abundance of 16SB and 16SA communities (Table 1). The OM removal %, ORR, N removal%, current production, and current density positively influenced the abundance of bacterial communities. The current production, power density, and current density had a negative influence on 16SA communities whereas they were positively influenced by OM removal % and ORR.

**Table 1:** Statistical results of the linear mixed effects models for physicochemical and electrochemical properties as controllers of changes in the absolute abundance of bacterial (16SB) and archaeal (16SA) communities

		16SB	16SA
OM removal %	Coefficient estimates, β	0.89	0.68
	Explained variance, R^2^ (%)	11.61	9.22
	Significance levels (*p*-value)	***	**
ORR	Coefficient estimates, β	0.77	0.66
	Explained variance, R^2^ (%)	15.22	7.84
	Significance levels (*p*-value)	***	**
N removal %	Coefficient estimates, β	0.55	−0.12
	Explained variance, R^2^ (%)	4.55	0.17
	Significance levels (*p*-value)	*	NS
pH anode	Coefficient estimates, β	0.11	0.12
	Explained variance, R^2^ (%)	0.23	0.09
	Significance levels (*p*-value)	NS	NS
Temperature anode	Coefficient estimates, β	0.15	0.22
	Explained variance, R^2^ (%)	0.04	0.09
	Significance levels (*p*-value)	NS	NS
Redox potential	Coefficient estimates, β	0.34	0.17
	Explained variance, R^2^ (%)	0.31	0.23
	Significance levels (*p*-value)	NS	NS
Suspended solids effluent	Coefficient estimates, β	0.16	0.34
	Explained variance, R^2^ (%)	0.04	0.19
	Significance levels (*p*-value)	NS	NS
Current production	Coefficient estimates, β	0.83	−0.59
	Explained variance, R^2^ (%)	19.7	6.78
	Significance levels (*p*-value)	***	*
Power density	Coefficient estimates, β	0.81	−0.66
	Explained variance, R^2^ (%)	21.4	6.43
	Significance levels (*p*-value)	***	**
Current density	Coefficient estimates, β	0.88	−0.65
	Explained variance, R^2^ (%)	23.33	4.95
	Significance levels (*p*-value)	***	**
	Treatment	**	**
	HRT	**	**
	TreatmentxHRT	**	**

All linear models fulfilled the normal distribution of the residuals (*p* > 0.37, Shapiro's test). Significant codes: *p* < 0.05, **p < 0.01, ***p < 0.001; NS, not significant. The explained variance (R^2^) of each predictor was calculated as sums of squares for each variable × 100 / sums of squares for all variables. Coefficient estimate (β) for each predictor is presented

### Diversity and composition of prokaryotic communities

**Fig. 4:**
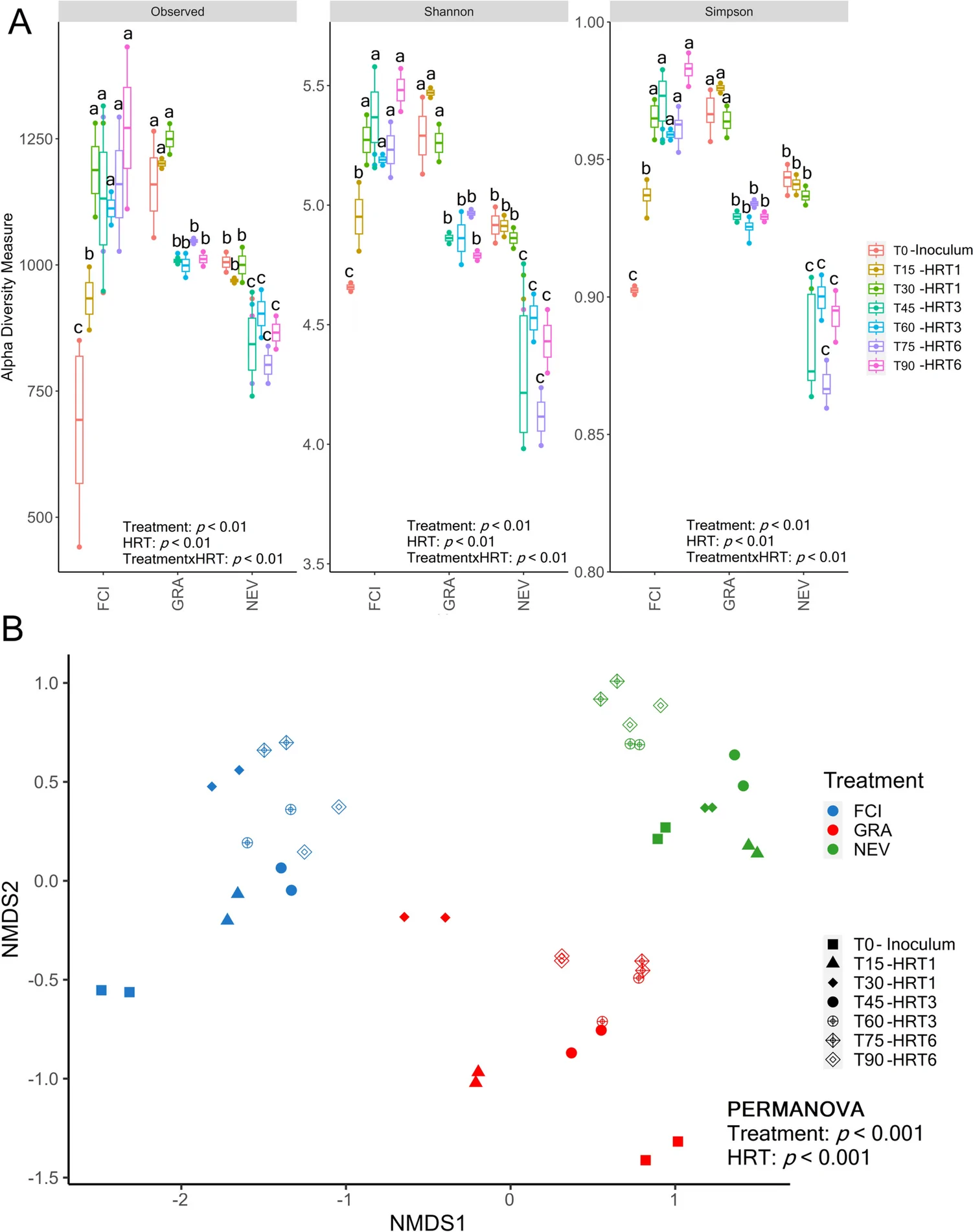
**A** Number of ASVs, and values of Shannon and inverse Simpson diversity indices for the prokaryotic community at different time points during the experimental period. Different letters above the bars indicate significant differences between time points. Linear mixed-effects model together with Tukey's post hoc test were used to look for significant differences between treatments and HRTs (*p* < 0.05). Values are expressed as mean with standard error. **B** Non-metric multidimensional scaling (NMDS) plots on unweighted UniFrac distances for the prokaryotic community at different time points during the experimental period. Differences in community composition between treatments and HRTs were tested by permutational analysis of variance (PERMANOVA). The MFC was inoculated with activated sludge from a fish-canning industry (FCI) and two different domestic WWPTs (GRA and NEV). Three consecutive HRTs were examined for each treatment: 1 day (HRT1), 3 days (HRT3), and 6 days (HRT6)

Treatment, HRT, and their interaction had a significant effect on the number of observed ASVs and the values of the Shannon and Simpson indices for the prokaryotic community (Fig. 2A). Treatment with FCI significantly increased alpha diversity until day 30 of operation at HRT1 to remain unchanged at HRT3 and HRT6 until the end of the experiment. When the MFC was treated with GRA and NEV, values of alpha diversity indices remained unchanged post-inoculation until day 30 of operation at HRT1. Then, they were significantly reduced at HRT3 and HRT6 towards the end of the experiment with no differences between these two HRTs. The beta diversity analysis revealed that treatment, HRT, and their interaction had a significant effect composition of the prokaryotic community (Fig. 4B). These variations in beta diversity were more pronounce at HRT1 compared to HRT3 and HRT6 for all treatments.

An overview of the prokaryotic community composition of the anode biofilm at the phylum and family taxonomic levels during the experimental period is shown in Supplementary Fig. S2. Generally, the anode biofilm of the MFC treated with FCI was dominated by taxa belonging to *Chloroflexi* and *Proteobacteria* phyla, and families such as *DEV007* and *A4b* during the whole experimental period. Phyla such as *Actinobacteria*, *Bacterioidota*, and *Proteobacteria* dominated in the MFCs treated with GRA and NEV. The most abundant families in the MFC treated with GRA were *Intrasporangiacea, Psedomononadaceae*, and *Comamonadaceae*. *Nocardiaceae, Cloacimonadaceae*, and *Sphingomonadaceaea* were the dominant families in the MFC treated with NEV.

### Differentially abundant prokaryotic taxa and their relationships with organic matter removal and current production

**Fig. 5:**
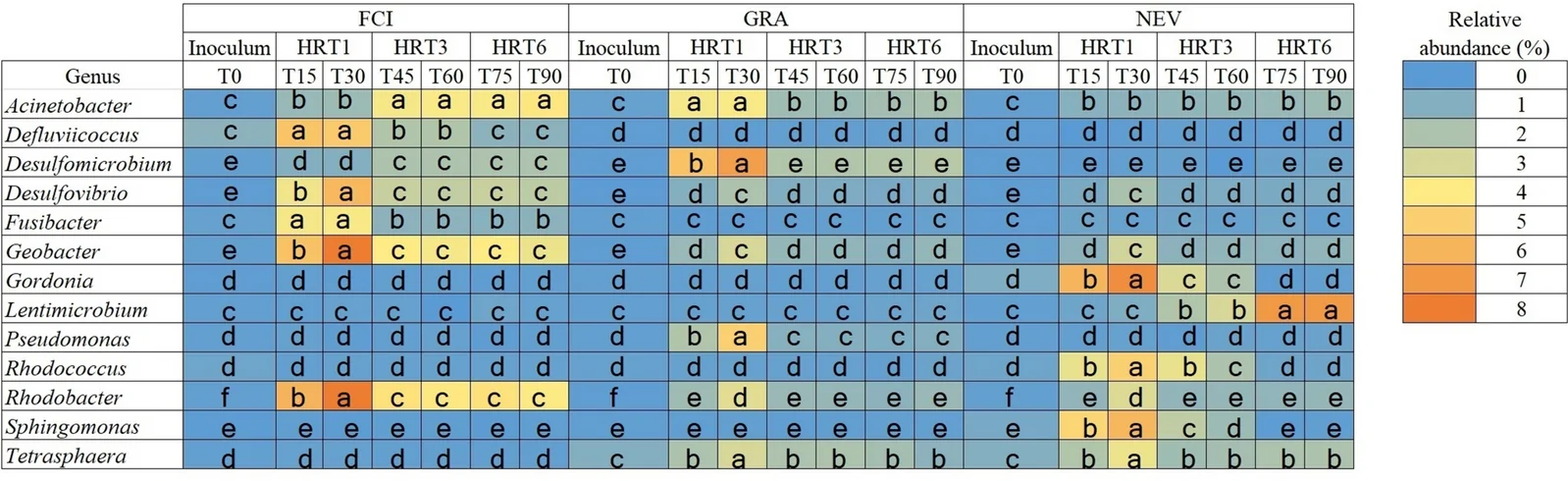
Heat map showing prokaryotic genera with relative abundance (%) significantly different between time points according to DESeq2 analysis. The MFC was inoculated with activated sludge from a fish-canning industry (FCI) and two different domestic WWPTs (GRA and NEV). Three consecutive HRTs were examined for each treatment: 1 day (HRT1), 3 days (HRT3), and 6 days (HRT6). Linear mixed-effects model together with Tukey's post hoc test were used to look for significant differences between HRTs and treatments (*p* < 0.05). For each genus, different letters denote significant differences between time points for all treatments

A total of 13 prokaryotic genera with significant differences in their relative abundance between time points were identified (Fig. 5). Among them, the *Defluviicoccus*, *Desulfovibrio*, *Geobacter*, and *Rhodobacter* were significantly enriched in the MFC treated with FCI compared to the GRA and NEV treatments. These increases were particularly greater at HRT1 compared to HRT3 and HRT6. *Desulfomicrobium* and *Pseudomonas* were significantly more abundant in the MFC treated with GRA at any HRTs compared to FCI and NEV treatments and values of relative abundance were significantly greater at HRT1 compared to HRT3 and HRT6. Treatment with NEV produced significant increases in the relative abundance of *Gordonia*, *Rhodococcus,* and *Sphingomonas* particularly at HRT1, and to a lesser extent at HRT3, compared to GRA and NEV treatments.

A Pearson correlation analysis revealed the relationships between the differentially abundant prokaryotic genera and OM removal % and current production (Supplementary Table S3). Significant positive correlations were observed between *Acinetobacter*, *Fusibacter*, *Lentimicrobium*, *Pseudomonas*, *Rhodococcus*, *Sphingomonas*, and *Tetrasphaera* and OM removal %. Current production was positively correlated with the relative abundance of *Defluviicoccus*, *Desulfomicrobium*, *Desulfovibrio*, *Fusibacter*, *Geobacter*, *Gordonia*, *Pseudomonas*, *Rhodococcus*, and *Rhodobacter*.

### Changes in predicted electron transfer genes

**Fig. 6:**
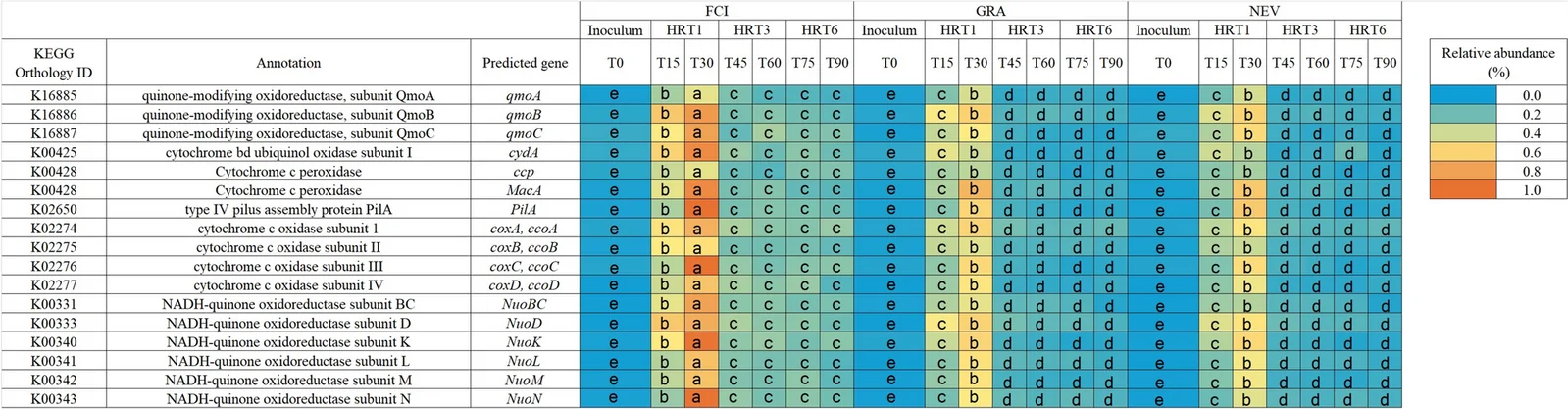
Heat map showing KEGG orthologs (KO) involved in the electron transfer pathway with significant differences in their relative abundance (%) between time points. The MFC was inoculated with activated sludge from a fish-canning industry (FCI) and two different domestic WWPTs (GRA and NEV). Three consecutive HRTs were examined for each treatment: 1 day (HRT1), 3 days (HRT3), and 6 days (HRT6). Linear mixed-effects model together with Tukey's post hoc test were used to look for significant differences between HRTs and treatments (*p* < 0.05). For each KO, different letters denote significant differences between time points for all treatments

Treatment and HRT had a significant effect on the relative abundance of 16 predicted KOs involved in electron transfer (Fig. 6). These KOs were associated with genes encoding different cytochromes b and c subunits, type IV pilus, NADH-quinones, and other quinone oxidoreductases. The relative abundance of the differentially abundant 16 KOs was significantly greater in FCI compared to GRA and NEV treatments for all time points, except in the inocula. Regardless of the treatment, the relative abundance of the predicted electron transfer KOs was significantly greater at HRT1 compared to HRT3 and HRT6. However, no significant differences in the relative abundance of the KOs were observed between HRT3 and HRT6 for all treatments.

## Discussion

This study demonstrates that the inoculum type and the HRT determine changes in the organic matter removal efficiency, bioelectricity generation, and the anodic microbiome of a continuous-flow MFC fed with saline wastewater. The inoculum from the fish canning industry significantly increased current generation and OM removal % compared to the inocula from domestic WWTPs. This effect was linked to a greater absolute and relative abundance of electroactive microorganisms (e.g., *Geobacter*, *Desulfovibrio*, and *Rhodobacter*) and predicted electron transfer genes in the anodic microbiome, likely due to better adaption to previous salinity conditions by microbial communities of the FCI inoculum. Regardless of the inoculum, our results revealed that the organic matter removal efficiency and current generation were enhanced at shorter HRT (1 day vs. 3 and 6 days). This effect was related to variations in the diversity, abundance, and composition of bacterial and archaeal communities in the anodic microbiome. For example, increases in OM removal % and energy production at HRT1 were linked to greater abundance and diversity of bacterial communities and increased number of KOs involved in electron transfer compared to longer HRTs. Limited bioelectricity generation was associated with reduced ORR and a greater abundance of archaeal communities, particularly at longer HRTs. Together, our findings have significant bioengineering implications, as they can help improve the performance of MFCs treating saline effluents, such as those from the seafood industry.

The inoculum type was found to determine variations in bioelectricity generation and OM removal efficiency in an MFC fed with saline wastewater and operated in continuous mode. Regardless of the HRT, the FCI inoculum led to the greatest values of voltage production, power and current densities, and ORR compared to inocula from domestic WWTPs. This could be due to the activated sludge from the fish-canning industry enriched with electroactive and non-electroactive microorganisms that may rapidly adapt to saline conditions in the wastewater. This assertion is supported by the rapid increases observed in the abundance of bacterial communities and the diversity of prokaryotic communities in the anodic microbiome following inoculation of the MFC with FCI, in contrast to GRA and NEV treatments. In general, the values of voltage production and power density in this study are higher than those reported in other studies using MFCs without saline effluents (Kim et al. 2015, 2016; Sobieszuk et al. 2017; Fazli et al. 2018; Ye et al. 2020). Previous research has demonstrated the potential of saline effluents to support bioenergy generation in MFCs due to their high conductivity (Lefebvre et al. 2012; Li et al. 2013; Guo et al. 2021). However, we extend those findings by showing inocula from industry effluents such as the fish canning industry can not only enhance bioelectricity generation but also the treatment performance of continuous-flow MFCs (You et al. 2010; Jayashree et al. 2016; Jamal and Pugazhendi 2021). Together, these results highlight the potential of continuous-flow MFC for practical applications.

Our findings also showed that the inoculum type determined changes in the composition of the anodic microbiome which was tightly linked to variations in OM removal % and current production. A group of known electroactive microorganisms such as *Defluviicoccus*, *Desulfovibrio*, *Fusibacter*, *Geobacter*, and *Rhodobacter* were enriched in the anode of the MFC inoculated with FCI compared to GRA and NEV treatments. All these genera contain species that are known to transfer electrons to anodes (Koch and Harnisch 2016; Ying et al. 2017; Castellano-Hinojosa et al. 2022a, b). The presence of these taxa was correlated with increased current production, thus suggesting inoculum selection is critical to enhance bioelectricity generation of continuous-flow MFC fed with saline wastewater. Wastewater from the seafood industry has been reported to be enriched with electroactive microorganisms. This is thought to be due to salinity favoring the growth of electroactive microorganisms compared to domestic wastewater (Guo et al. 2021; Xin et al. 2022). Notably, a total of 16 predicted KOs involved in electron transfer (e.g., cytochromes, pilus, and quinones) in the anodic microbiome were identified, which were significantly more abundant in the MFC inoculated with FCI compared to GRA and NEV treatments. Together, these results suggest that inoculum selection not only determines differences in the diversity and composition of the anode microbiome but also in its functionality, all of which are critical traits for efficient electron transfer.

It is interesting to note that there were differences in the abundance, diversity, and composition of prokaryotic communities between GRA and NEV treatments which could be related to the location of the domestic WWTPs. For example, the NEV inoculum was collected from a WWTP located in a high-mountain area above 2000 m a.s.l. whereas the GRA inoculum was taken from a WWTP in Granada city (about 670 m a.s.l.). Previous studies have shown that temperature can impact the microbial ecology of activated sludge in WWTPs (Jung and Regan 2007; Griffin and Wells 2016). Our findings suggest that differences in microbial ecology between inocula from domestic WWPTs can determine variations in the composition of electroactive microorganisms which may subsequently impact the performance of MFCs. For example, *Desulfomicrobium*, *Pseudomonas*, and *Rhodobacter* were the most abundant exoelectrogenic microorganisms in the MFC treated with GRA whereas *Gordonia* and *Rhodoccocus* were the dominant electroactive bacteria after treatment with NEV.

Regardless of the inoculum, HRT was found to determine changes in the efficiency of removal of organic compounds and energy production, an effect that appears to be also related to variations in the anodic microbiome. The ORR was increased at shorter HRT (1 day vs. 3 and 6 days) when the ORL was higher. These results can be explained by a lower concentration of organic compounds for use by exoelectrogenic microorganisms at lower OLR, which can result in lower ORR and decreased current generation. This agrees with other studies that showed decreases in power density with reduced OLR at longer HRTs (Liu et al. 2008; Sharma and Li 2010; Ye et al. 2020; Castellano-Hinojosa et al. 2024). Jamal and Pugazhendi (2021) reported that inoculation of MFCs with halophilic consortium can achieve OM removal % of about 84% (initial concentration of 1.21 g COD L^−1^) at 20 days of HRT. Here, it was detected an OM removal % of about 65% at an HRT of only 6 days with double the initial COD (2.46 g COD L^−1^) when the MFC was inoculated with FCI. Together, these results suggest that activated sludge from the seafood industry could be a suitable inoculum for enhancing both organic removal and bioenergy generation in MFCs treating saline effluents.

Increases in OM removal % and voltage production at HRT1 were linked to greater abundance and diversity of bacterial communities, along with an increased number of KOs involved in electron transfer, compared to longer HRTs. This could be due to the shorter HRT favoring fast colonization of the anode and subsequent growth of microbial communities, as supported by qPCR results and predicted functionality. Interestingly, it was also noted that limited bioelectricity generation was correlated with an increased abundance of archaeal communities, particularly evident at longer HRTs. Previous studies have shown that the proliferation of archaeal communities at longer HRTs can reduce bioelectricity generation in MFCs, an effect that has been related to competition between exoelectrogenic microbes and methanogens for electrons (Castellano-Hinojosa et al. 2024). Alternative processes such as direct or mediated interspecies electron transfer (IET and MIET, respectively) between electroactive bacteria and archaea within the anode can also diminish voltage production (Quéméner et al. 2021). We acknowledge that our findings were obtained using synthetic wastewater and real effluents may contain more complex organic compounds than the acetate used in this study. Future studies should explore the impact of inoculum selection and HRT on bioenergy generation and prokaryotic communities of MFCs treating real wastewater. Nonetheless, our findings remain useful as all comparisons in this study were performed under similar operational conditions (e.g., influent composition, temperature, HRT, etc.).

This study shows that the inoculum type and the HRT can impact the removal efficiency of organic compounds, bioelectricity generation, and the anodic microbiome of a continuous-flow MFC fed with saline wastewater. Such insights hold the potential for enhancing the valorization of saline effluents from both industrial and domestic wastewater treatment plants (WWTPs) through continuous-flow MFC technology. Our findings show that activated sludge from the fish canning industry increased the electricity generation and organic matter removal compared to the inocula from domestic WWTPs. These effects were tightly linked to variations in the anodic microbiome at different HRTs. For example, the inoculum from the seafood industry favored greater absolute and relative abundances of electroactive microorganisms and predicted electron transfer genes likely due to better adaption to salinity conditions by microbial communities of this inoculum type. Additionally, it was found that the removal efficiency of organic compounds and current generation is enhanced at shorter HRTs (1 day vs. 3 and 6 days) regardless of the inoculum type. This effect was linked to changes in the abundance, diversity, and composition of bacterial and archaeal communities in the anodic microbiome.

Despite our findings, scaling up H-cell MFC technology for industrial use still presents several key challenges that should be addressed in future studies. For instance, the construction and maintenance of large-scale reactors involve significant costs, especially in saline environments that require corrosion-resistant materials (Prathiba et al. 2024). Cost-effective and life cycle assessment studies are needed to compare the suitability of continuous-flow MFCs for treating saline effluents versus other existing technologies. Operational challenges, such as maintaining stable and efficient microbial populations over time, are also critical, as fluctuations in microbial communities could impact treatment performance and electricity generation. Moreover, shorter HRTs, while beneficial for efficiency, may increase operational complexity and costs. Addressing these challenges will require advancements in reactor design, such as improved materials that reduce costs and enhance durability. Additionally, developing strategies to maintain stable microbial populations, such as optimizing inoculum types and environmental conditions, will be crucial. The potential for bioelectricity generation offers an economic incentive, which could be maximized by integrating MFC systems with existing industrial processes. Collaborative efforts across research, industry, and policy will be essential to overcoming these barriers and realizing the full potential of H-cell MFC technology in practical applications.

## Supplementary Information

Below is the link to the electronic supplementary material.

Supplementary file1 (PDF 459 KB)

## Data Availability

Data will be made available on request.
