# Supplementary material for: Inoculum selection and hydraulic retention time impacts in a microbial fuel cell treating saline wastewater
Source: Appl Microbiol Biotechnol. 2025 Jan 28;109:29. doi: 10.1007/s00253-024-13377-y (PMC11774983; doi:10.1007/s00253-024-13377-y)
Supplement: Supplementary file 1 [file 253_2024_13377_MOESM1_ESM.pdf]

Supplementary Material

Inoculum selection and hydraulic retention time impacts in a microbial fuel cell treating saline wastewater

Authors: Antonio Castellano-Hinojosa[1,2]*, Manuel J. Gallardo-Altamirano[1,3], Clementina Pozo[1,2], Alejandro González-Martínez[1,2], Jesús González-López[1,2]

Affiliations:

[1]Environmental Microbiology Group, Institute of Water Research, University of Granada, 18003, Granada, Spain

[2]Department of Microbiology, University of Granada, 18071, Granada, Spain

[3]Department of Chemical Engineering, University of Granada, 18071, Granada, Spain

*Corresponding author: Tel: +34 958248321. Email: ach@ugr.es (Antonio Castellano-Hinojosa)

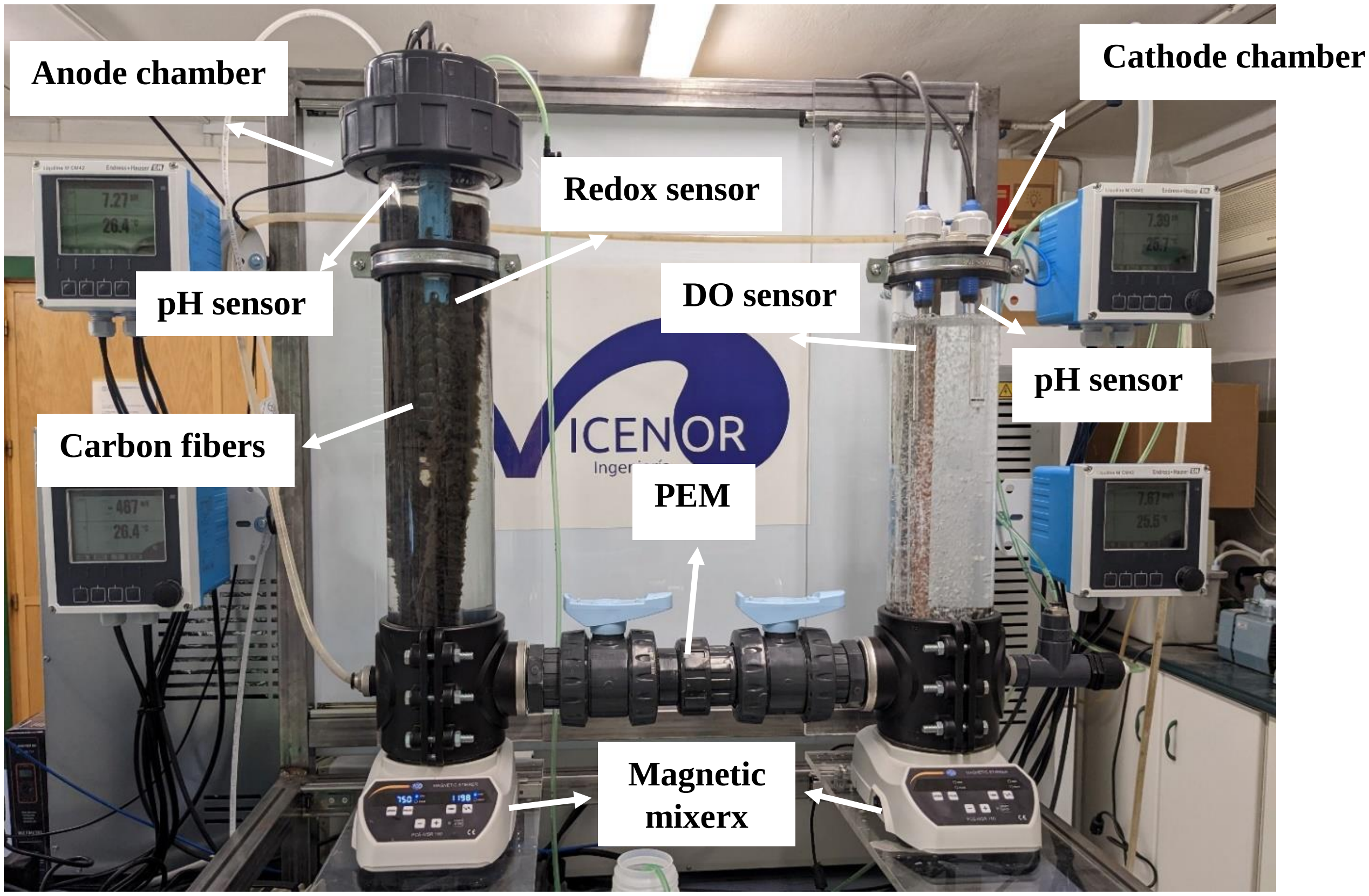

Supplementary Fig. S1. A picture of the microbial fuel cell (MFC) used in this study. PEM, proton exchange membrane; T, temperature; DO, dissolved oxygen

Supplementary Table S1. Primers used for quantification of the absolute abundance of bacterial and archaeal communities (16SB and 16SA, respectively) by qPCR (A). Bacterial and archaeal strains used for generation of qPCR standards are also included. qPCR conditions for the quantification of each of the target genes (B).

A.

Primer	Primer sequence (5´-3´)	Target gene	Strains	Reference
341F	CCTACGGGAGGCAGCAG	16S rRNA Bacteria	*Pseudomonas putida* NCB957	Muyzer et al. (1993)
534R	ATTACCGCGGCTGCTGG			
ARCH915F	AGGAATTGGCGGGGGAGCAC	16S rRNA Archaea	Genomic clone 29i4	Yu et al. (2008)
UNI-b-revR	GACGGGCGGTGTGTRCAA			

B.

	16SB	16SA
Stage 1: 1 cycle	3 min at 95 ºC	7 min at 95 ºC
Stage 2: 40 cycles	15s at 95 ºC	30s at 95 ºC
	30s at 60ºC	30s at 65 ºC
	30s at 72 ºC	30s at 72 ºC
Stage 3: 1 cycle	10 min at 72 ºC	10 min at 72 ºC

Supplementary Table S2. Changes in the pH, temperature, and redox potential in the anode, suspended solids in the effluent, and N removal efficiency (%) between the influent and effluent during the experimental period. The MFC was inoculated with activated sludge from a fish-canning industry (FCI) and two different domestic WWPTs (GRA and NEV). Three consecutive HRTs were examined for each treatment: 1 day (HRT1), 3 days (HRT3), and 6 days (HRT6). Values are expressed as mean with standard error. Linear mixed-effects model together with Tukey's post hoc test were used to look for significant differences between HRTs and treatments ($p < 0.05$). For each column, values followed by the same letter are not statistically different for each treatment.

A.

FCI						
HRT	Day of operation	pH anode	Temperature anode (ºC)	Redox potential anode (mV)	Suspended solids effluent (mg L^{-1})	N removal (%)
HRT1	1	7.6 ± 0.2a	21.3 ± 0.3a	-246 ± 12a	31.5 ± 2.1a	29.6 ± 2.2a
	5	7.7 ± 0.1a	21.2 ± 0.2a	-357 ± 8a	27.3 ± 3.9ab	28.1 ± 2.0a
	8	7.8 ± 0.1a	21.2 ± 0.2a	-467 ± 7a	24.2 ± 2.1b	28.8 ± 1.9a
	11	7.7 ± 0.2a	21.3 ± 0.1a	-480 ± 6a	20.1 ± 1.8b	28.2 ± 2.3a
	14	7.8 ± 0.1a	21.3 ± 0.2a	-477 ± 5a	15.2 ± 1.9c	27.5 ± 1.7a
	21	7.8 ± 0.2a	21.1 ± 0.1a	-478 ± 8a	12.1 ± 2.7c	30.4 ± 1.8a
	30	7.8 ± 0.2a	21.4 ± 0.2a	-480 ± 5a	13.3 ± 1.9c	31.2 ± 2.6a
HRT3	32	7.9 ± 0.2a	21.5 ± 0.2a	-485 ± 6a	14.3 ± 2.2c	28.4 ± 3.2a
	37	8.0 ± 0.1a	21.6 ± 0.3a	-481 ± 4a	13.5 ± 1.5c	29.2 ± 2.5a
	40	8.0 ± 0.1a	21.5 ± 0.2a	-473 ± 5a	12.5 ± 2.4c	29.4 ± 2.4a
	44	7.8 ± 0.1a	21.6 ± 0.1a	-475± 8a	13.6 ± 1.7c	27.7 ± 2.6a
	49	7.9 ± 0.2a	21.2 ± 0.2a	-476 ± 5a	12.8 ± 1.5c	29.9 ± 1.9a
	53	8.0 ± 0.1a	21.3 ± 0.3a	-475 ± 5a	12.9 ± 2.0c	28.5 ± 2.5a
	60	8.0 ± 0.2a	21.4 ± 0.3a	-476 ± 7a	11.6 ± 1.6c	29.5 ± 2.2a
HRT6	62	8.0 ± 0.2a	21.6 ± 0.2a	-489 ± 5a	13.6 ± 2.0c	26.3 ± 3.0a
	66	8.1 ± 0.2a	21.3 ± 0.1a	-480 ± 7a	12.5 ± 2.3c	27.5 ± 2.7a
	72	8.0 ± 0.1a	21.6 ± 0.2a	-488 ± 5a	12.5 ± 2.8c	28.2 ± 1.8a
	77	8.0 ± 0.1a	21.4 ± 0.3a	-475 ± 6a	12.5 ± 2.4c	29.4 ± 3.2a
	81	7.9 ± 0.1a	21.3 ± 0.2a	-467 ± 6a	12.7 ± 3.0c	28.2 ± 3.2a
	85	7.8 ± 0.2a	21.5 ± 0.2a	-470 ± 7a	13.9 ± 2.3c	29.5 ± 1.9a
	90	7.9 ± 0.2a	21.2 ± 0.2a	-477 ± 8a	12.7 ± 2.5c	26.5 ± 2.1a

B.

GRA						
HRT	Day of operation	pH anode	Temperature anode (ºC)	Redox potential anode (mV)	Suspended solids effluent (mg L^{-1})	N removal (%)
HRT1	1	7.3 ± 0.2a	21.6 ± 0.3a	-252 ± 16a	24.1 ± 2.4a	33.6 ± 3.2a
	5	7.3 ± 0.1a	21.4 ± 0.2a	-334 ± 11a	14.3 ± 3.1b	35.1 ± 3.4a
	8	7.2 ± 0.1a	21.5 ± 0.2a	-477 ± 10a	12.2 ± 2.2bc	37.8 ± 3.4a
	11	7.3 ± 0.2a	21.3 ± 0.1a	-483 ± 7a	12.1 ± 2.5c	38.2 ± 3.3a
	14	7.3 ± 0.1a	21.5 ± 0.2a	-486 ± 7a	11.2 ± 1.3c	37.5 ± 3.7a
	21	7.2 ± 0.2a	21.4 ± 0.1a	-488 ± 8a	10.1 ± 2.1c	36.4 ± 2.5a
	30	7.1 ± 0.2a	21.3 ± 0.2a	-484 ± 8a	11.3 ± 1.2c	34.2 ± 2.6a
HRT3	32	7.3 ± 0.2a	21.3 ± 0.2a	-489 ± 6a	12.3 ± 2.7c	39.4 ± 3.5a
	37	7.5 ± 0.1a	21.6 ± 0.3a	-489 ± 7a	11.5 ± 1.4c	37.2 ± 2.3a
	40	7.4 ± 0.1a	21.3 ± 0.2a	-481 ± 5a	11.5 ± 2.2c	36.4 ± 2.5a
	44	7.3 ± 0.1a	21.2 ± 0.1a	-480± 6a	10.6 ± 2.0c	35.7 ± 2.6a
	49	7.3 ± 0.2a	21.2 ± 0.2a	-483 ± 6a	10.8 ± 1.8c	38.9 ± 2.4a
	53	7.3 ± 0.1a	21.4 ± 0.3a	-485 ± 8a	9.9 ± 2.7c	35.5 ± 2.5a
	60	7.1 ± 0.2a	21.2 ± 0.3a	-483 ± 7a	12.6 ± 1.4c	36.5 ± 2.2a
HRT6	62	7.3 ± 0.2a	21.6 ± 0.2a	-482 ± 5a	11.6 ± 2.1c	37.3 ± 3.4a
	66	7.2 ± 0.2a	21.5 ± 0.1a	-480 ± 8a	9.5 ± 2.1c	39.5 ± 2.7a
	72	7.1 ± 0.1a	21.6 ± 0.2a	-494 ± 5a	10.5 ± 2.2c	35.2 ± 2.4a
	77	7.5 ± 0.1a	21.5 ± 0.3a	-483 ± 9a	9.5 ± 2.1c	36.4 ± 3.6a
	81	7.4 ± 0.1a	21.3 ± 0.2a	-477 ± 6a	11.7 ± 2.7c	37.2 ± 3.2a
	85	7.3 ± 0.2a	21.3 ± 0.2a	-474 ± 6a	11.9 ± 2.2c	36.5 ± 2.5a
	90	7.3 ± 0.2a	21.3 ± 0.2a	-474 ± 5a	10.7 ± 2.2c	35.5 ± 2.7a

C.

NEV						
HRT	Day of operation	pH anode	Temperature anode (ºC)	Redox potential anode (mV)	Suspended solids effluent (mg L^{-1})	N removal (%)
HRT1	1	7.2 ± 0.1a	21.2 ± 0.2a	-254 ± 11a	18.5 ± 2.1a	35.2 ± 2.5a
	5	7.1 ± 0.1a	21.4 ± 0.2a	-378 ± 11a	13.2 ± 3.0b	32.5 ± 3.2a
	8	7.2 ± 0.1a	21.2 ± 0.2a	-474 ± 12a	8.1 ± 2.2c	32.6 ± 2.7a
	11	7.1 ± 0.1a	21.3 ± 0.2a	-478 ± 8a	8.1 ± 2.7c	31.5 ± 3.2a
	14	7.2 ± 0.1a	21.2 ± 0.2a	-476 ± 7a	8.5 ± 1.3c	33.5 ± 2.8a
	21	7.2 ± 0.2a	21.4 ± 0.2a	-471 ± 9a	8.1 ± 2.6c	34.7 ± 2.2a
	30	7.2 ± 0.1a	21.2 ± 0.2a	-469 ± 6a	7.8 ± 1.9c	32.2 ± 2.9a
HRT3	32	7.2 ± 0.2a	21.3 ± 0.2a	-474 ± 6a	8.3 ± 2.7c	33.4 ± 3.1a
	37	7.1 ± 0.1a	21.2 ± 0.1a	-472 ± 7a	8.5 ± 2.6c	35.7 ± 2.5a
	40	7.2 ± 0.2a	21.3 ± 0.2a	-471 ± 7a	7.6 ± 2.7c	32.4 ± 2.5a
	44	7.2 ± 0.1a	21.1 ± 0.1a	-467± 6a	8.6 ± 2.0c	33.5 ± 2.8a
	49	7.2 ± 0.2a	21.2 ± 0.1a	-479 ± 6a	7.6 ± 2.2c	34.9 ± 2.9a
	53	7.1 ± 0.2a	21.3 ± 0.3a	-478 ± 8a	8.4 ± 2.7c	31.8 ± 2.3a
	60	7.2 ± 0.2a	21.2 ± 0.3a	-466 ± 5a	8.3 ± 1.9c	33.5 ± 2.2a
HRT6	62	7.2 ± 0.2a	21.3 ± 0.2a	-477 ± 5a	8.6 ± 2.1c	34.3 ± 3.2a
	66	7.2 ± 0.1a	21.5 ± 0.2a	-472 ± 6a	7.9 ± 2.5c	33.8 ± 2.2a
	72	7.5 ± 0.1a	21.2 ± 0.2a	-472 ± 5a	8.5 ± 2.2c	35.2 ± 2.4a
	77	7.2 ± 0.2a	21.5 ± 0.1a	-467 ± 6a	8.5 ± 2.4c	33.5 ± 3.2a
	81	7.2 ± 0.1a	21.2 ± 0.2a	-479 ± 6a	7.9 ± 2.3c	33.4 ± 3.1a
	85	7.3 ± 0.2a	21.1 ± 0.1a	-478 ± 6a	9.0 ± 2.3c	34.1 ± 2.2a
	90	7.2 ± 0.2a	21.3 ± 0.2a	-477 ± 5a	8.3 ± 2.2c	35.1 ± 2.5a

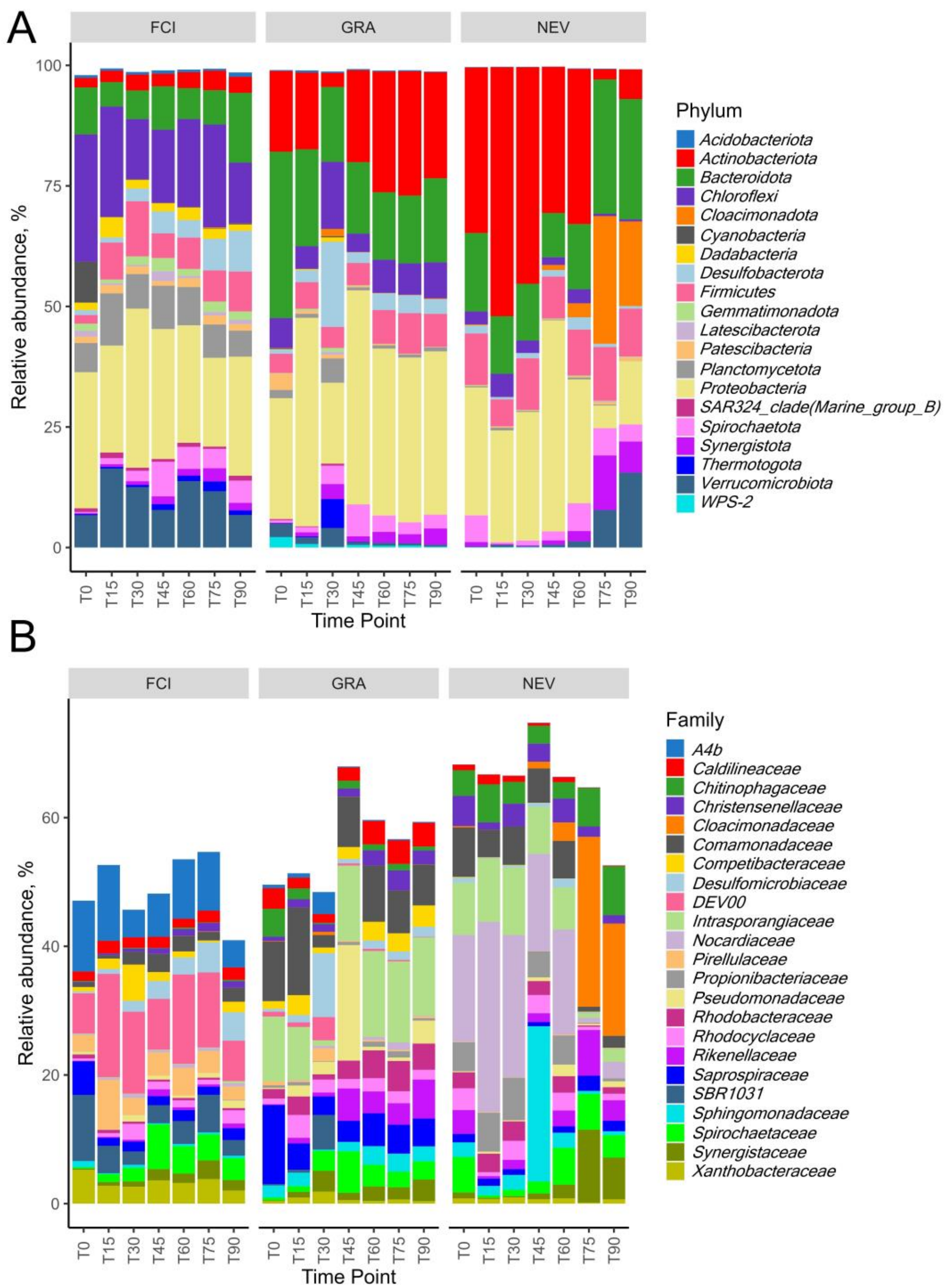

Supplementary Fig. S2. Relative abundance of prokaryotic ASVs at the phylum (A) and family (B) taxonomic levels during the experimental period. The MFC was inoculated with activated sludge from a fish-canning industry (FCI) and two different domestic WWPTs (GRA and NEV). Three consecutive HRTs were examined for each treatment: 1 day (HRT1), 3 days (HRT3), and 6 days (HRT6). Phyla and families with more than 0.5 and 1% of relative abundance are shown, respectively.

Supplementary Table S3. Pearson correlation coefficients between the relative abundance of the differentially abundant genera, OM removal %, and current production. Significant correlations are boldface.

Genus	OM removal %	Current production
Acinetobacter	**0.85**	0.25
Defluviicoccus	0.52	**0.61**
Desulfomicrobium	0.27	**0.69**
Desulfovibrio	0.33	**0.86**
Fusibacter	**0.84**	**0.85**
Geobacter	0.32	**0.92**
Gordonia	0.24	**0.68**
Lentimicrobium	**0.85**	0.25
Pseudomonas	**0.65**	**0.78**
Rhodococcus	**0.62**	**0.86**
Rhodobacter	0.26	**0.77**
Sphingomonas	**0.84**	0.42
Tetrasphaera	**0.84**	0.45

References

Muyzer, G., De Waal, E.C., Uitterlinden, A.G., 1993. Profiling of complex microbial populations by denaturing gradient gel electrophoresis analysis of polymerase chain reaction amplified genes coding for 16S rRNA. Appl. Environ. Microbiol. 59, 695-700.

Yu, Z., García-González, R., Schanbacher, F.L., Morrison, M., 2008. Evaluation of different hypervariable regions of archaeal 16S rRNA genes in profiling of methanogens by archaea-specific PCR and denaturing gradient gel electrophoresis. Appl. Environ. Microbiol. 74, 889-893.
